# A Study of the Radiation Tolerance and Timing Properties of 3D Diamond Detectors

**DOI:** 10.3390/s22228722

**Published:** 2022-11-11

**Authors:** Lucio Anderlini, Marco Bellini, Vladimir Cindro, Chiara Corsi, Keida Kanxheri, Stefano Lagomarsino, Chiara Lucarelli, Arianna Morozzi, Giovanni Passaleva, Daniele Passeri, Silvio Sciortino, Leonello Servoli, Michele Veltri

**Affiliations:** 1 National Institute for Nuclear Physics of Florence, Sesto Fiorentino, 50019 Florence, Italy; 2 National Institute of Optics-CNR of Florence, Sesto Fiorentino, 50019 Florence, Italy; 3 LENS—European Laboratory for Non-Linear Spectroscopy of Florence, Sesto Fiorentino, 50019 Florence, Italy; 4 Department of Physics and Astronomy, University of Florence, Sesto Fiorentino, 50019 Florence, Italy; 5 Jozef Stefan Institute, 1000 Ljubljana, Slovenia; 6 Department of Physics and Geology, University of Perugia, 06100 Perugia, Italy; 7 National Institute for Nuclear Physics of Perugia, 06123 Perugia, Italy; 8 Laboratory of Nano-Optics, University of Siegen, 57076 Siegen, Germany; 9 Engineering Department, University of Perugia, 06123 Perugia, Italy; 10 Department of Pure and Applied Sciences, University of Urbino, 61029 Urbino, Italy

**Keywords:** CVD diamond, diamond sensors, laser engineering, timing measurements, radiation hardness

## Abstract

We present a study on the radiation tolerance and timing properties of 3D diamond detectors fabricated by laser engineering on synthetic Chemical Vapor Deposited (CVD) plates. We evaluated the radiation hardness of the sensors using Charge Collection Efficiency (CCE) measurements after neutron fluences up to 10${}^{16}$ n/cm${}^{2}$ (1 MeV equivalent.) The radiation tolerance is significantly higher when moving from standard planar architecture to 3D architecture and increases with the increasing density of the columnar electrodes. Also, the maximum applicable bias voltage before electric breakdown increases significantly after high fluence irradiation, possibly due to the passivation of defects. The experimental analysis allowed us to predict the performance of the devices at higher fluence levels, well in the range of 10${}^{16}$ n/cm${}^{2}$. We summarize the recent results on the time resolution measurements of our test sensors after optimization of the laser fabrication process and outline future activity in developing pixel tracking systems for high luminosity particle physics experiments.

## 1. Introduction

Radiation tolerance and high spatial and temporal resolution are the most sought-after characteristics of an ionizing radiation sensor [1]. Diamond has long been studied as the base material for the fabrication of sensors in various fields [2,3] including experiments in high energy physics [4], clinical dosimetry [5], the detection of neutrons [6], and deep UV radiation [7,8], due to its strong chemical bond (which in principle, implies a high resistance to radiation compared to silicon [9], the standard base material of solid-state detectors) and other favorable characteristics. The high saturation speed and the high breakdown field are important characteristics for timing detectors; the low dark current (pa/cm^2^) and the lower dielectric constant than silicon are factors that increase the signal-to-noise ratio. It is noteworthy that diamond sensors do not require cooling even after maximum irradiation, being very capable of dissipating heat (diamond has a high thermal diffusivity and the highest thermal conductivity of any solid material), while also operating well above ambient temperature [10] or in the presence of ambient light, unlike silicon. On the other hand, the charge generated by a relativistic particle (minimum ionizing particle, MIP) in diamond is significantly lower than that of silicon, and also depends on the quality of the samples [11]; in addition, the charge collected can be strongly degraded by crystalline defects. A synthetic diamond (chemical vapor deposited, CVD) can only be grown into wafers in its polycrystalline form (pCVD), which exhibits low efficiency, typically half the charge signal of a sensor made from single-crystal (scCVD) diamond. Homoepitaxially monocrystalline crystalline diamond is limited, at present [12], to areas of the order of 1 cm^2^ and the fact that there are very few suppliers makes the cost prohibitive. Recently, considerable progress has been made in the synthesis of (almost) monocrystalline material, grown heteroepitaxially in 3.6” wafers [13]. The electronic quality of this material still seems significantly lower than that of the best single crystals due to structural defects (dislocations) [14]. The 3D architecture of the electrodes of the solid-state detectors has been proposed to increase the radiation tolerance of silicon sensors [15]. This is conceived to maintain the same active volume as the standard planar architecture, while decreasing the distance between the electrodes by an order of magnitude, so that the charge carriers are less sensitive, in their path to the readout eletrodes, to the crystalline defects, whether pristine or induced by radiation. Secondly, the 3D architecture allows much faster response time provided that the geometry of the electrodes is suitably designed to overcome the drawback of a highly non-uniform field [16,17].

We have demonstrated, in the past [18], the capability of 3D monocrystalline diamond sensors to detect MIPs, using a laser micro-machining technique [19] based on multi-photon excitation in the femtosecond regime [20]. The columnar electrodes of the devices, fabricated in the diamond bulk, were interconnected by surface graphitization, using a nanosecond laser [21].

We have subsequently shown that the radiation resistance of polycrystalline diamond detectors with 3D architecture is significantly higher than that of detectors with standard planar contacts [22]. In this work, we extend the study of the radiation tolerance of detectors developed on monocrystalline diamond showing that the radiation resistance strongly increases with decreasing the electrode distance.

Other than for their radiation hardness, small-pitch 3D diamond pixel detectors are particularly appealing for their potentially excellent time resolution. This aspect has been recently studied by the Timespot collaboration [23], aiming to develop complete prototypes of tracking devices, based on silicon and diamond 3D pixel detectors, and compare them in terms of their temporal resolution and their radiation hardness, for applications at future accelerator experiments.

Homoepitaxial monocrystalline diamond currently seems to be the only possible choice for the construction of diamond timing sensors, due to the quite lower signal-to-noise ratio attained with other CVD diamond base material. In this work, we summarize the progress made in optimizing the timing resolution of 3D detectors fabricated on the monocrystalline diamond, starting from the initial 280 ps [24,25,26] up to close to the performance of silicon detectors [27]. We also outline the expected developments of the experiment in the near future.

## 2. Materials and Methods

The samples used in this study are all synthetic “electronic grade” CVD monocrystalline diamond plates, produced by Element Six Ltd. (Ascot, UK). They exhibit a charge collection efficiency (CCE) for MIPs quite close to 100% (an ideal efficiency, as inferred from Hecht’s theory [28]) probably thanks to a minimum presence of substitutional or aggregate impurities (<1 ppb [29]). All samples are of similar same size 4.5–5 × 4.5–5 mm^2^ and the nominal thickness is 500 μm. The two faces are optically polished to allow laser processing for sensor fabrication. Some of the side facets were polished for optical inspection of the electrodes fabricated into the bulk.

**Figure 1:**
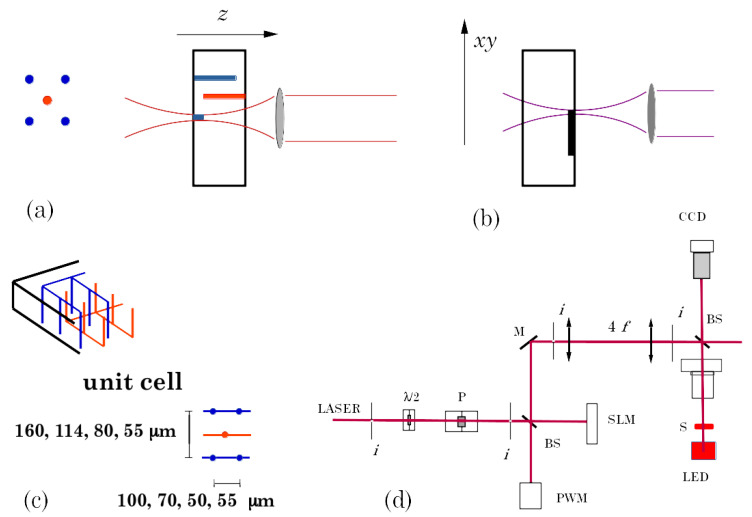
Fabrication process schematics. (**a**) We write the conductive columns in the bulk of the diamond by translating the fs laser beam waist along the optical axis (z), i.e., along the thickness of the sample. (**b**) We connect the electrodes for implementing test structures by writing graphitic paths on the diamond surface with ns laser irradiation. (**c**) The figure shows the interpenetrating matrices of bulk electrodes (bias columns and signal columns). It also shows the unit cell sizes implemented (see text.) (**d**) The figure shows an oversimplified description of the optical setup. We align the laser beam through the irises (*i*). The beam passes through a rotatable λ/2 lamina and a polarizer (P), from which we control the input power via the power meter (PWM), then it is collimated on the spatial light modulator (SLM.) Upon reflection, it is collected by a 4f-system, then focused with a high numerical aperture objective on the sample (S), mounted on a *xyz* stage. A computer controls all the steps of the process via python codes.

The schematics of Figure 1 describe the whole process of sensors’ fabrication.

Columnar electrodes were manufactured by irradiation with a Ti: Sa laser at 800 nm, mode-locked with a pulse duration of about 50 fs, the precise value depending on the optics each time interposed between the laser and the target. We did not detect any particular dependence of the properties of the electrodes on this value. The phase transition from $sp^{3}$ to $sp^{2}$ bond occurs through multi-photon excitation and therefore requires a well-defined focus with a high numerical aperture objective and the correction of aberrations in real-time through a spatial light modulator (Hamamatsu LCOS-SLM X10468-02) [30]. Although the columnar electrode is opaque to an optical inspection, the modified material cannot be pure microcrystalline graphite, for thermodynamic reasons, due to the lower density of the graphite phase, which gives rise to very high-pressure fields [21], but a weakly connected mixture of $sp^{3}$ and $sp^{2}$ phases [31]. The sensors used for timing measurements were implemented by writing two matrices of interpenetrated columns. The columns of each matrix start from one face (bias or signal) and are terminated at about 50 μm from the opposite face, to avoid surface discharges between electrodes at different voltages.

Electrode fabrication with ns laser would result in considerable damage to the crystal lattice, while this pulse duration is more suitable for surface graphitization because the graphite material formed on the surface is not removed as in the case of the fs laser [21]. For this reason, we used a 1064 nm, 8 ns, Q-switched Nd: YAG laser to connect several unit cells in test sensors [22].

The test structures for the study of radiation damage have 100 × 160 μm, 70 × 114 μm, 50 × 80 μm unit cells. The structures used for timing measurements have a unit cell of 55 × 55 μm.

**Figure 2:**
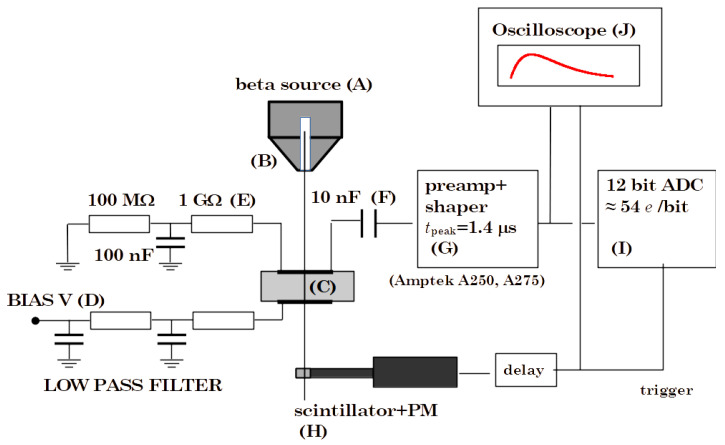
Charge CollectionEfficiency measurement setup developed by Fred Hartjes (NIKHEF, Amsterdam). (**A**), beta source, (**B**), collimator, (**C**), device under test, (**D**), power supply, (**E**), 1 GΩ load resistor, (**F**), decoupling capacitor, (**G**), Amptek shaper, (**H**), trigger, (**I**), PCI NI ADC Board, (**J**) oscilloscope.

We performed the radiation tolerance study using Sr-90 sources and a measurement setup sketched in Figure 2 and fully described in Ref. [32]. Pulse height spectra were acquired for all test structures irradiated at neutron fluences up to $\phi =10^{16}$/cm^2^ (1 MeV equivalent). The CCE is empirically defined here as the ratio between the charge collected at the electrodes and the calculated value of the charge generated in the active volume of the sample (between the planar contacts or within the unit cell in the case of 3D detectors). The irradiations were carried out at the Triga reactor in Ljubljana, under the care of the Josef Stefan Institute. The test structures are wire-bonded to the readout electronics. It should be noted that our test samples are all-carbon sensors and therefore it was not necessary to reconstruct the contacts after each irradiation as reported in other radiation hardness studies [9].

**Figure 3:**
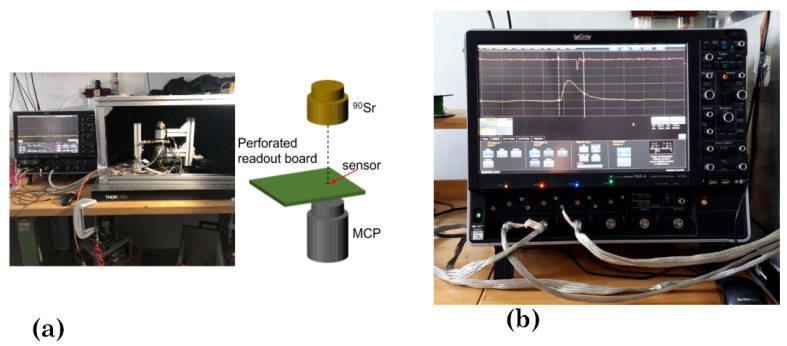
Laboratory setup for timing measurements. (**a**) Overview with a schematic of the aligned system of excitation (a beta source) and triggering (MCP-PMT). (**b**) Detail of the measurement: signals from the diamond test structure and the reference, displayed by the oscilloscope.

Test sensors prepared for timing measurements were measured with a laboratory system outlined in Figure 3. Beam tests were then carried out at CERN in Geneva. The laboratory system for timing tests uses a 10 mCi Sr-90 source and a laser sample alignment system mounted on the reading board and reference sensor. The reading board was designed by Kansas University and described in [33]. The reference sensor is an MCP-PMT Photonis (Hi QE UV FT8, PP2365Z) with a temporal resolution of 20 ps. The waveforms of the sensor under test and reference are recorded using a LeCroy WavePro 760Zi-A oscilloscope (6 GHz, 40Gs/s). The time markers are evaluated offline from the signal waveforms via an algorithm of constant fraction discrimination (CFD).

A similar system was used in the beam test.

The beam test was carried out on the 180 GeV SPS-H8 98 pion beam at CERN, selecting minimum ionizing pions through the coincidences between the signal from a PHOTONIS MCP-PMT and the signal from a 55 × 55 μm Timespot silicon pixel sensor. The time resolution of the trigger system was measured to be about 20 ps. The position of the silicon pixel was controlled with a Newport picomotor piezo platform, corresponding to a small region on the diamond sensor, of the order of a few μm. The signals were acquired with a Rhode–Schwartz oscilloscope, operated at 20 GS/s.

In this article, we compare the current results of sensors optimized in terms of laser fabrication system optics and bulk electrode resistance with those of a previous Villigen PSI beam test [26]. For the upcoming final Timespot beam test, we prepared 32 × 32 pixel detectors with a 55 μm pitch, bump-bonded by Fraunhofer IZM of Berlin to a dedicated electronics chip developed by the Timespot collaboration.

## 3. Results

### 3.1. Radiation Hardness Study

We irradiated the diamond samples at the experimental nuclear reactor of the Jozef Stefan Institute of Ljubljana (SLO), with fast neutrons (neutrons of energy greater than 100 keV). The hardness factor of the neutron spectra has been evaluated considering the non-ionizing energy loss (NIEL) of neutrons in the range of 100 keV–30 MeV, normalized to the NIEL of neutrons at 1 MeV [34]. Table 1 shows the irradiation fluences used (n/cm^2^, 1 MeV equivalent).

The best estimate we can give of the fluence value ϕ is within a ten percent error based on the reactor power measurement. It is the same for low and high fluences since the irradiation interval was maintained long enough, and therefore the time uncertainty was kept low, by lowering the power of the reactor at low fluences. The *CCE* of the test sensors was measured before and after every irradiation. A quartz plate was interposed between the sample and the trigger scintillator–photomultiplier assembly to select only the events produced by the MIP particles from the Sr-90 source, passing through both 500 μm of diamond and 1 mm of quartz. The G gain of the *CCE* station was determined with both a test capacitor and a silicon diode, in full depletion. The two measures are compatible and give G = (240 ± 10) *e*/mV. With this G value, two of our monocrystalline samples, measured with planar ohmic contacts, gave a signal at saturation, with an applied field of about 0.4 V/μm, of 20,500 *e* (thickness 505 μm) and 22,000 *e* (thickness 505 μm), respectively. Assuming the generation rate (number of electron–hole pairs generated per unit length) N = 43.1 e/μm, as reported in [14], for a MIP in high-quality scCVD diamond, we derive about a 95% CCE for our samples in planar configuration at a 200 V bias voltage.

We first highlight two notable results of this series of measures.

It is known that a procedure of priming deep diamond traps [35] is required, by irradiating charged particles or photons, in certain frequency ranges [36], also referred to as “pumping”. After this process, the sensor shows a higher efficiency which is maintained for some time, if the sample is not heated or illuminated with light in a “depumping” wavelength range. We performed the priming process with a 10 mCi Sr-90 source before measurement for which a 0.1 mCi Sr-90 was adequate. We have observed that the higher quality samples, i.e., the monocrystalline ones, needed a very small dose of priming or did not need it at all. Intuitively, the increase in efficiency in the “pumped state” is less and less relevant as the crystalline quality increases, i.e., the priming efficiency anti-correlates with the *CCE* [37]. As already noted in [22], the required priming dose was very different for non-irradiated and irradiated samples and increased with increasing levels of irradiation. Moreover, 5 Gy was sufficient in the first case, for a standard "electronic grade" pCVD sample, while the order of 100 Gy it was necessary for the latter, for any type of sample. We also noted a polarization effect [37], that is, the lowering of the collected charge during the measurement, due to a distortion of the local field inside the bulk (while the bias voltage is kept constant). This effect is due to a differential charge trapping inside the diamond bulk; therefore, we primed the samples, before measurement, at zero applied bias. Furthermore, to minimize the effect, during the acquisition of the pulse height spectrum, the bias voltage was frequently reversed.

**Figure 4:**
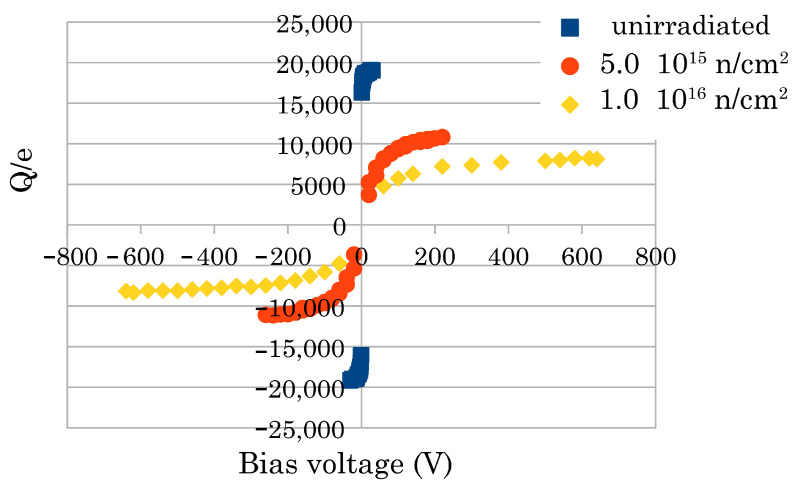
Measured charge vs. bias voltage for the 500 col/mm^2^ sensor (S-curves). We expressed the charge in the number of electrons, i.e., normalized the collected charge value by the elementary charge *e*. The allowed maximum voltage increases with the irradiation fluence (see text).

**Figure 5:**
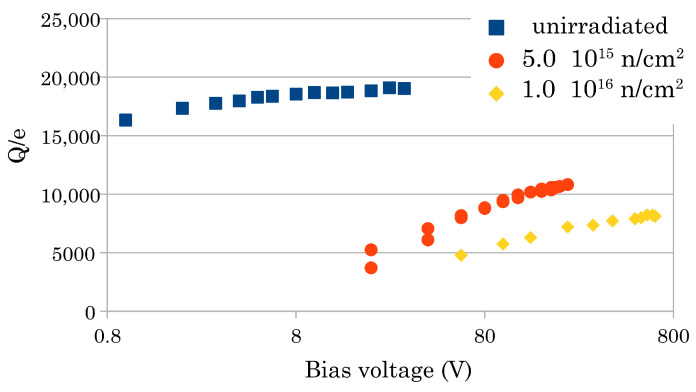
S-curves of the 500 col/mm^2^ sensor in semi-log scale.

Second, we recently observed that after a fluence of 5 × 10^15^ n/cm^2^, we could use a higher bias voltage (without the electrical breakdown observed in the non-irradiated sample). At 10^16^/cm^2^ we could use 640 V with the finest structured sensor (500 columns/mm^2^). Figure 4 shows the S-curves of the collected charge of that sensor highlighting the extension of the bias voltage range as the fluence increases. Interestingly, as shown in Figure 5, detailing the same curves in a semi-log scale, we have a signal of more than 8000 electrons, at that irradiation level, at 640 V (the corresponding sigma noise we measure is about 500 *e*). After this fluence, the 2D sensors yield a signal below 700 *e*.

**Figure 6:**
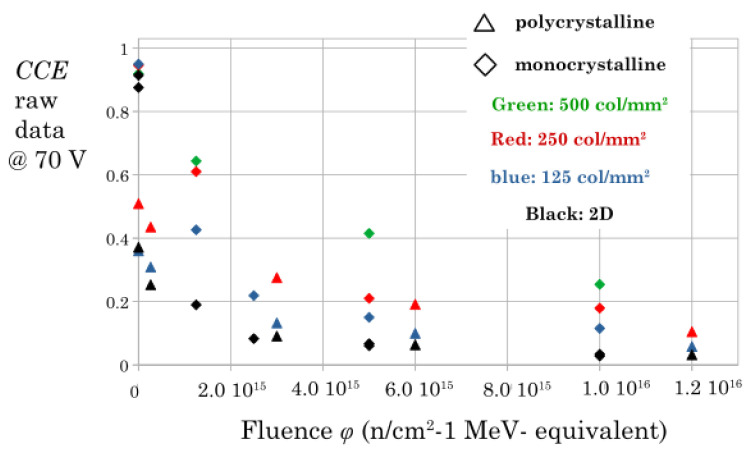
Measured charge collection efficiencies for all the samples at a 70 V bias voltage (see text). The markers are diamonds for the scCVD sensors and triangles for the pCVD material. Different colors denote different geometries as noted in the legend.

Figure 6 shows the whole dataset of the measured efficiencies at a bias voltage *V* = 70 V. This bias value is typically the maximum allowed for our unirradiated 3D sensors. Above this value, we observe an electric breakdown after which the bias leakage current increases exponentially and the corresponding noise makes it impossible to operate the sensor. This effect is completely reversible. Furthermore, this effect quenches as the radiation damage increases.

To fit the experimental data, we need to express the charge collection distance *CCE* as a function of the irradiation fluence. CCE will depend on the effective lifetime, τ, of the charge carriers: $CCE=f(\frac{1}{\tau })$. The τ parameter is related to traps in the diamond bandgap, which are governed by crystalline, pristine, and radiation-induced defects. We have adopted the empirical relationship between τ and ϕ [9]:

(1) $$\frac{1}{\tau }=\frac{1}{\tau_{0}}+k\phi ,$$

where $\tau_{0}$ is a measure of the quality of the undamaged sample and *k* is usually termed as the “damage constant”. Using Equation (1), we can express the efficiency of a sensor in terms of the irradiation fluence: $f\left(\frac{1}{\tau }\left(\phi \right)\right)=F\left(\phi \right)$ In the case of 2D planar architecture the function *f* is derived by the Hecht formula [9]:

(2) $$CCE=2\times \left\{\;\frac{\lambda }{L}-\frac{\lambda^{2}}{L^{2}}\left[1-\exp\left(-\frac{L}{\lambda }\right)\right]\right\},$$

Factor two takes into account the contribution of both holes and electrons, assuming the same effective mean free path λ. The parameter λ is related to the mean lifetime of the charge carriers by the relation λ=v(E)τ, where v(E) is the drift velocity, which depends on the applied field intensity *E*. We adopted the empirical expression used by several authors to best fit the drift velocity experimental data [38,39]:

(3) $$v\left(E\right)=\frac{\mu E}{1+\mu E/v_{s}},$$

where $v_{s}$ is the saturation velocity and μ is the small field mobility. It has been reported [40] that there are some disagreements on the mobility values in the comparison between different experimental measurement techniques, possibly also dependent on a certain variability in the quality of the samples in the various studies. Nonetheless, it seems reasonable to choose values of μ = 2000 cm^2^/(V · s) for the mobility of both carriers. We also set the saturation velocity value as $v_{s}=2\times 10^{7}$ cm/s according to some reported studies [38]. The velocity then is calculated at any field, i.e., at any bias voltage, using the chosen $v_{s}$ and μ values.

In the case of the 3D planar architecture, the field is not uniform, and we have to calculate $f\left(\frac{1}{\tau }\right)=F\left(\phi \right)$ for the different geometries of our sensors. To this purpose, we wrote a code to simulate the charge induced by a beam of particles passing through a unit cell, utilizing a Monte Carlo algorithm, using the Ramo–Shockley theorem [22]. The electric field was pre-determined at each point of the unit cell employing a three-dimensional finite element calculation (Synopsys Sentaurus TCAD).

Finally, we fitted all the experimental data with the calculated CCE curves using $\tau_{0}$ and *k* as fit parameters. The prominent result of our analysis is that we could best fit all our data with a unique value of *k*, as previously reported in another study (only on planar sensors) [9], i.e., the damage constant depends only on the crystalline nature of the material whether monocrystalline or polycrystalline.

**Figure 7:**
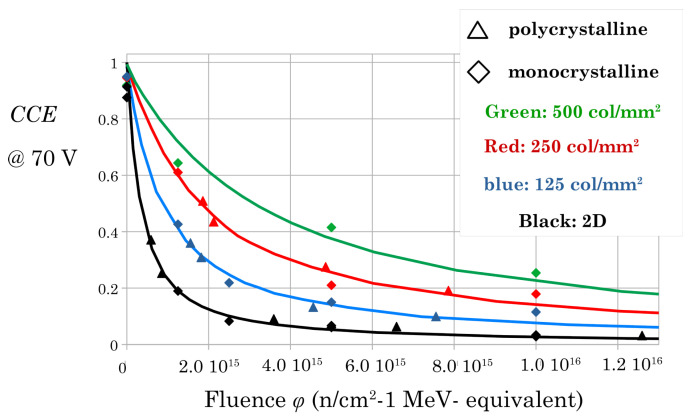
Experimental data fitted by the calculated *CCE* curves with a unique damage factor *k*.

Furthermore, we could arrange all the experimental data in one single plot by considering the initial inefficiency of the unirradiated samples as an effective fluence $\phi_{0}$ i.e., using the formal position: $1/\tau_{0}+\phi =\phi_{0}+\phi$, where $\phi_{0}$ is different for different samples and different geometries. The final plot is presented in Figure 7 where one can interpret the lower fluence of any marker as the effective $\phi_{0}$. That is, the experimental values of the unirradiated samples are shifted horizontally by $\phi_{0}$ with respect to Figure 6. Note that we could set $\phi_{0}=0$ for all the monocrystalline samples because their starting inefficiency is very small.

### 3.2. Timing Study

**Figure 8:**
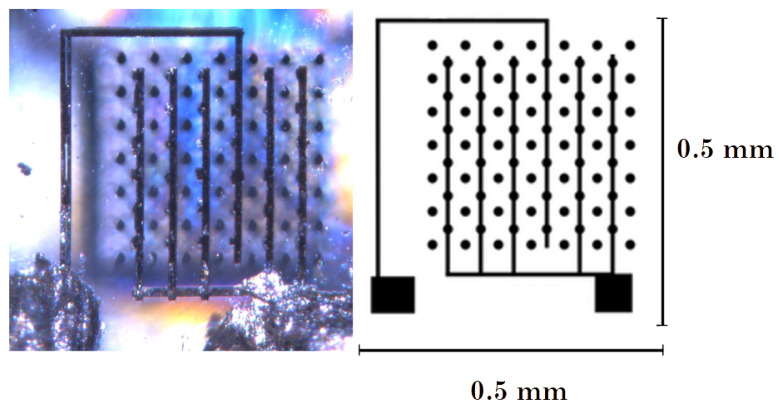
Test structure on an scCVD diamond (unit cell: 55 × 55 μm) used for the timing measurements both in the laboratory and under the beam.

**Figure 9:**
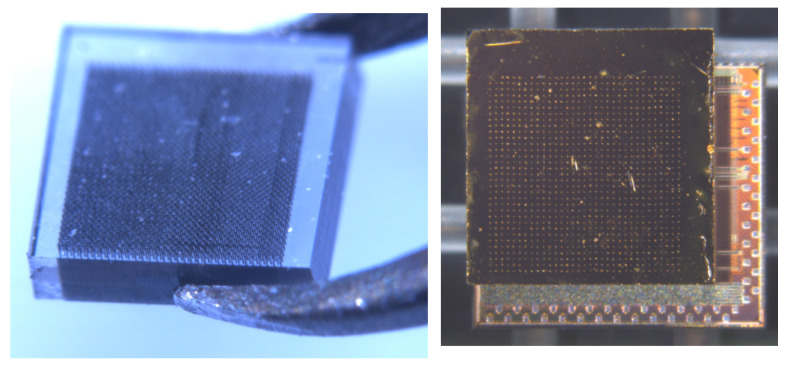
(**Left**): 32 × 32 pixel sensor fabricated on a 2.3 × 2.3 mm^2^ scCVD sample. (**Right**): the same sample bump-bonded to the Timespot chip for future beam tests.

Figure 8 shows the test structure used for both laboratory measurements and the most recent beam test [41]. It was implemented after the completion of the optimization of the laser micro-fabrication process, resulting in a decrease in the resistance of the columnar electrodes from hundreds of kΩ down to about 30 kΩ (with an electrode diameter of about 10 μm). The signal side is clearly visible with a strip and a comb of columns connected with surface graphite lines, together with the bias columns, all short-circuited, starting from the opposite side and buried in the bulk. Figure 9 shows the column matrix built on a 2.3 × 2.3 mm sample, prepared as a 32 × 32 pixel sensor and the same sensor bump-bonded on the Timespot ASIC. We have prepared 8 of these sensors to characterize them and to implement a diamond telescope for an upcoming beam test.

We briefly summarize, below, the updated status of our experimental results and compare them with theoretical simulations.

#### 3.2.1. Laboratory Tests

The test structure was fully characterized with the laboratory setup, the detailed analysis is reported in [42]. The most relevant features of the laboratory test are two. First, we observed a noticeable charge-sharing effect, because a significant amount of charge was generated by the MIP in more than one unit cell. This was due to the divergence of the electrons from the source, the large multiple scattering affecting the electrons, and the aspect ratio—the ratio of the linear size of the unit cell to the thickness of the sample. This effect could be corrected by adding the coincidence signals of both the single strip and the comb. No clear peaks were visible in the pulse height spectrum of the strip; however, by summing the charge released in neighboring cells, measured coincidentally with the comb channel, a noticeable Landau peak clearly emerges from the noise. The same was true for the comb spectrum, albeit with a slightly worse S/N ratio, due to the higher capacitance.

The time resolution is provided by the delay distribution width. The delay is defined as the time difference between the time markers set on the signal by the diamond sensor and the MCP-PMT, used in the trigger system.

A crystal ball fit of the single-strip delay distribution resulted in global temporal resolution $\sigma_{TOT}\approx$ 120 ps (the influence of MCP-PMT is almost negligible).

Time resolution as a function of signal amplitude follows a simple model [43] quite well:

(4) $$\sigma_{t}=\sqrt{\sigma_{a}^{2}+{\left(\sigma_{noise}\cdot \bar{t_{r}}\right)}^{2}/A^{2}}$$

where $\sigma_{noise}$ is the average electronic noise, $\bar{t_{r}}$ is the average signal rise time, A is the signal amplitude and $\sigma_{a}$ is the asymptotic resolution in absence of noise. The latter is found as the limit for large signals which turns out to be $\sigma_{a}\approx 75$ ps.

#### 3.2.2. Beam Test

Contrary to laboratory tests with the beta Sr-90 source, the beam test allowed data acquisition in a well-defined region within the sensitive area of the sensor.

**Figure 10:**
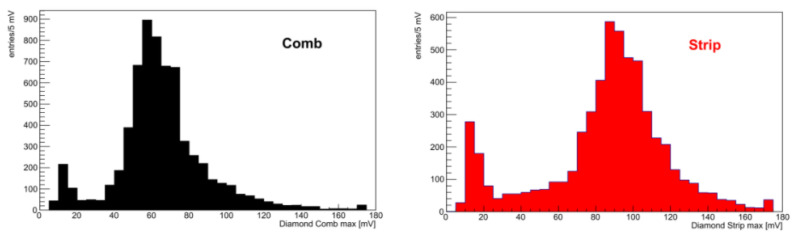
(**Left**): pulse height spectrum of the comb test structure. (**Right**): pulse height spectrum of the strip test structure.

**Figure 11:**
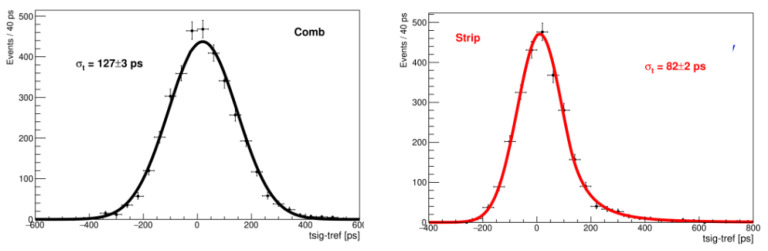
(**Left**): delay distribution of the comb test structure. (**Right**): delay distribution of the strip test structure.

Figure 10 shows the amplitude distributions for the strip and the comb, both structures showing a clear Landau peak, with an S/N ratio of 12 for the comb and 18 for the strip. The delay distribution for the comb (Figure 11) is symmetrical and corresponds to the temporal resolution of $\sigma_{t}=127\pm 3$ ps, determined by a Gaussian fit. The distribution of the strip delay is narrower and results in a time resolution of 82 ± 2 ps.

**Figure 12:**
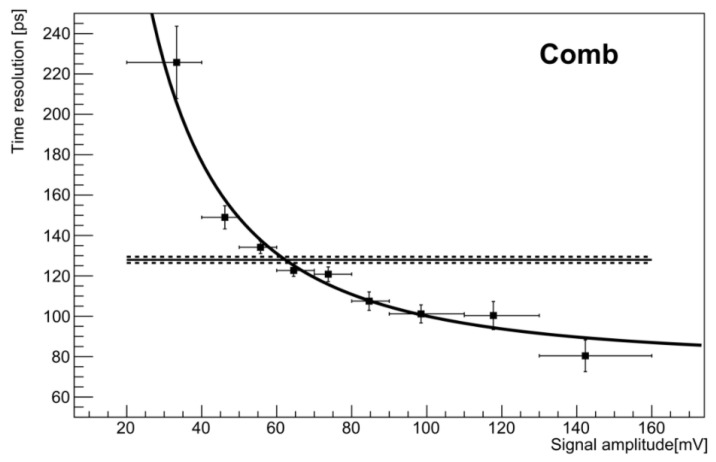
Time resolution in bins of signal amplitude; the line is the fitted simplified resolution model of equation [43]. The horizontal line represents the resolution value resulting from the delay distribution of Figure 11 (left).

The asymptotic behavior at large signals is represented for the comb in Figure 12. The fit according to the simple model of Equation (5) produced a value $\sigma_{a}=72$ ps.

#### 3.2.3. Comparison of Experimental Data with Simulations

**Figure 13:**
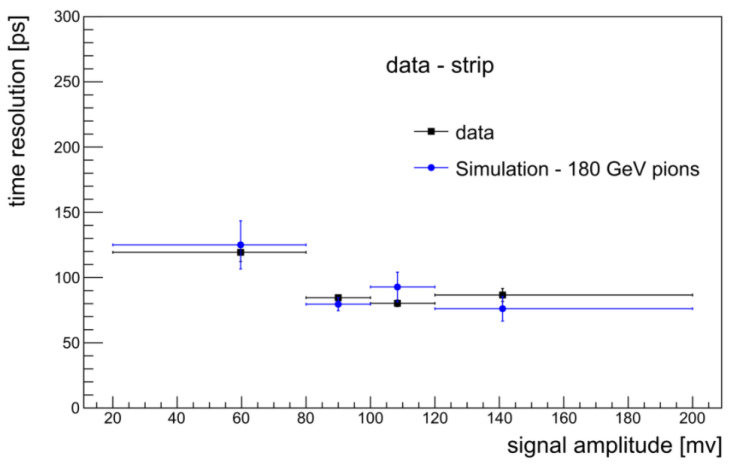
Time resolution as a function of signal amplitude for data (black squares) and simulated (blue circles) 180 GeV pion beam.

We investigated the mechanism of signal formation in 3D diamond sensors, from the charge generation to the output signal, through simulations. First, we use Geant4 to simulate the passage of MIP particles inside the detector. We focus on the signal produced by one single pixel and to account for the charge flow from and to the surrounding pixels we model our detector as a 3 × 3 pixel matrix traversed by the incident particles. Following the tracks inside the detector, step by step, the amount and position of the produced ionization charge are stored in a file for later use. The so-generated charge carriers are then drifted in the electric field to the electrodes using the ROOT-based simulator KDetSim. This is a low CPU usage program providing a fast simulation of semiconductor detectors assumed to be in a steady state. The resistive readout electrodes are treated as capacitive transmission lines and the propagation of the induced signal currents to the detector surface along the line and finally to the electronics is modeled by convolving the induced signal with the response function of the line and the readout board. To complete the procedure, real noise taken from data is added to the simulated waveforms, which are subsequently analyzed with the same algorithms used for real data. The simulation process has been validated with both the laboratory data source [42] and beam test data [41]. Figure 13 shows the comparison of the time resolution as a function of the signal amplitude for real and simulated data. A good agreement can be observed indicating the overall correctness of our approach.

## 4. Discussion

Our radiation damage model relies on the choice of parameters, such as mobility and saturation rate, which find disagreement in the literature. For this reason, our damage factor value is affected by a significant bias. However, a single value $k=\left(1.5\pm 0.15\right)\times 10^{-6}$ cm${}^{2}$ s${}^{-1}$ (statistical error) can consistently account for all our experimental data for both monocrystalline and polycrystalline samples. This determination allows us to predict the relative decay of the signal at higher irradiation levels.

We characterized, in the past, polycrystalline electronic-grade CVD material, correlating different efficiencies of the sensors with variations in the concentration of deep traps [35].

On the other hand, evidence of decreased leakage current or increased rise time due to reduction in defect concentration by neutron irradiation has been reported in the past [44,45]. This latter effect is also a prominent result of the present investigation: the shift of the voltage threshold of the electric breakdown of an order of magnitude is likely due to the passivation of defects involved in conduction.

**Figure 14:**
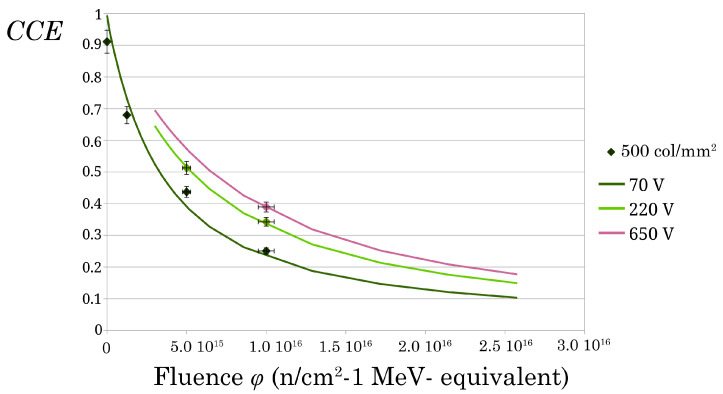
Measured and simulated CCE at increasing bias voltages, for the 500 col/mm${}^{2}$ 3D sensor. We plotted the experimental points until the highest voltage level allowed by our power supply. The CCE curves tend to zero as fluence increases (and lifetime decreases). Nonetheless, a high irradiation level permits to increase in the bias voltage, so the sensors are operative in the 10${}^{16}$ n/cm${}^{2}$ range.

According to our data analysis, we can push the irradiation level further by simulating, as shown in Figure 14, the behavior of the finest pitch sensor (500 col/mm^2^, 50 × 80 μm unit cell), which exhibited the highest radiation tolerance, at different polarization conditions.

The detector is still operating after fluences well in the 10^16^ n/cm^2^ range and the signal can probably be increased by applying higher voltages before breakdown. Furthermore, the diamond radiation tolerance can be still improved by a substantial decrease in the unit cell size.

In previous works, the RD42 CERN collaboration has extensively studied the radiation tolerance of diamond detectors. Ref. [9] reports comprehensively on the results relative to planar sensors. The results for neutron irradiation agree with the present study, yielding a mean free path of tens of micrometers after the highest irradiation. The damage factor is calculated by use of the relation:

(5) $$\frac{1}{\lambda }=\frac{1}{\lambda_{0}}+K^{\prime }\phi$$

Since the planar geometry assumes field uniformity, the mean free path λ can be directly related to the charge collection efficiency via Hecht’s Formula (2), so the analysis is not affected by the uncertainty on the fit constants discussed above.

On the other hand, we used our model relatively to predict future irradiation results to be verified experimentally. The relevant finding of our analysis is the dramatic relative increase in radiation hardness with the increasing granularity of the 3D electrodes.

Coming to the timing application, a substantial decrease in the modified material resistivity is still needed to come closer to the 3D silicon performances. It is not clear at present which is the limiting value in the case of columnar growth along the diamond thickness. The lowest value reported [30] is ρ=0.022 Ωcm (±5f%), against our 0.4–0.5 Ωcm; however, this record value is obtained with laser modification in the bulk parallel to the sample faces, i.e., under a condition where the aberration correction is made once and for all.

**Figure 15:**
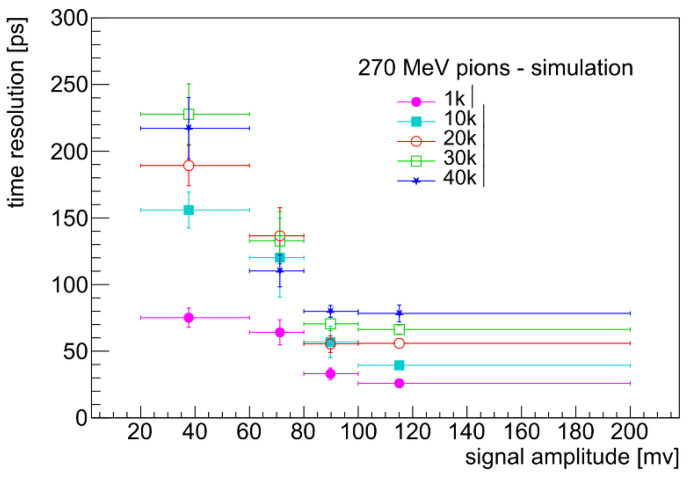
Time resolution as a function of signal amplitude for simulated 270 MeV pions, for different values of the electrode resistance.

The striking agreement between our simulation procedure and the experimental data allows us to predict the improvement in resolution, by a decrease of one order of magnitude of the resistance value, from the present tens of kΩ to 1 kΩ. The improvement is significant as shown in Figure 15. More research and development on diamond laser engineering is needed to determine that resistance value through a substantial decrease in resistivity.

## Figures and Tables

**Table 1:** Table of the irradiation fluences ϕ (n/cm^2^, 1 MeV equivalent). The new irradiations (scCVD samples) are indicated in the right column. Those carried out before this work [22] (pCVD samples) are on the left.

Fluence ϕ (n/cm^2^), pCVD Samples [22]	Fluence ϕ (n/cm^2^), scCVD Samples
2.6 × 10^14^	
	1.25 × 10^15^
	2.5 × 10^15^
3.0 × 10^15^	
	5.0 × 10^15^
6.0 × 10^15^	
	1.0 × 10^16^
1.2 × 10^16^	

## Data Availability

The data presented in this study are available on request from the corresponding author.
